# Layer-by-Layer-Processed All-Polymer Solar Cells with Enhanced Performance Enabled by Regulating the Microstructure of Upper Layer

**DOI:** 10.3390/molecules29122879

**Published:** 2024-06-17

**Authors:** Yixuan Wu, Peng Li, Shiqi Yu, Yonggang Min, Liangang Xiao

**Affiliations:** School of Materials and Energy, Guangdong University of Technology, Guangzhou 510006, China

**Keywords:** layer-by-layer, all-polymer solar cells, additive, morphology control, charge recombination

## Abstract

The layer-by-layer (LBL) fabrication method allows for controlled microstructure morphology and vertical component distribution, and also offers a reproducible and efficient technique for fabricating large-scale organic solar cells (OSCs). In this study, the polymers D18 and PYIT-OD are employed to fabricate all-polymer solar cells (all-PSCs) using the LBL method. Morphological studies reveal that the use of additives optimizes the microstructure of the active layer, enhancing the cells’ crystallinity and charge transport capability. The optimized device with 2% CN additive significantly reduces bimolecular recombination and trap-assisted recombination. All-PSCs fabricated by the LBL method based on D18/PYIT-OD deliver a power conversion efficiency (PCE) of 15.07%. Our study demonstrates the great potential of additive engineering via the LBL fabrication method in regulating the microstructure of active layers, suppressing charge recombination, and enhancing the photovoltaic performance of devices.

## 1. Introduction

Organic solar cells (OSCs), noted for their light weight, solution processability, and compatibility with flexible substrates, have become a focal point for researchers worldwide [1,2,3,4,5,6,7]. Currently, state-of-the-art single-junction OSCs have achieved a power conversion efficiency (PCE) exceeding 19% [8,9,10]. High-efficiency OSCs primarily employ the bulk heterojunction (BHJ) structure, which is spontaneously formed via phase separation from a mixed solution of electron donors and acceptors during the film deposition process. To optimize the microstructure’s morphology, researchers have developed a range of strategies, such as thermal annealing (TA), solvent vapor annealing (SVA), and additive engineering [11,12,13,14]. However, the photovoltaic performance of BHJ OSCs is highly dependent on molecular crystallinity, molecular orientation, phase separation, and vertical phase distribution within the active layer, which is often unpredictable and sensitive to material properties and processing conditions. This unpredictability within their fabrication creates challenges in the scaling up for large-scale industrial production and limits their potential for commercial applications.

In recent years, the layer-by-layer structure, fabricated by depositing electron donor and acceptor in separate solutions, has allowed for controlled microstructure morphology and vertical component distribution, and also offers a reproducible and efficient technique for fabricating large-scale OSCs [15,16,17]. During the LBL deposition process, the interfacial diffusion of electron donors and acceptors can lead to the formation of a p-i-n type structure within the active layer [18,19,20]. Such LBL-type devices are valued for their ease of reproducibility and reduced carrier recombination. By incorporating a wax additive to form nanoscale pores within the PM6 layer, a novel interdigitated heterojunction structure based on PM6/L8-BO bilayer heterojunction OSCs was successfully fabricated via the LBL deposition method. This novel structure resulted in an ideal vertical phase distribution inside the active layer, leading to an efficient exciton diffusion length and dissociation, and reduced charge recombination [21]. By adjusting the solution’s temperature and the annealing processes, the D18 polymer’s pre-aggregation behavior in solution can be controlled, leading to the manipulation of the microstructure of the D18 bottom layer. This optimized bottom layer effectively facilitates the formation of suitable networks in the L8-BO upper layer for efficient charge transport and deliver an enhanced PCE of 18.02% [22].

In the array of methods for regulating microstructure morphology of active layer, the use of additives stands out as a straightforward yet potent method to regulate the molecular packing, enhance the crystalline, and improve the blend film morphology [23,24]. For the study of the additives, it is essential to delve deeper into the mechanisms how these additives influence on the film morphology evolution. For example, the introduction of a selective solvent as an additive can swell the donor domains and improve the donor–acceptor interfacial area, facilitating more efficient charge separation and extraction processes. In addition to the chemical interactions, the physical properties of additives such as their boiling point and evaporation rate can also be critical. Solvent additives may remain in the film for a longer time during the film formation process, facilitating the molecules’ self-assemble into a well-structured microstructure morphology [25]. The reported studies have demonstrated the effectiveness of using an additive to fine-tune the morphology of the active layer, resulting in a more ordered structure and a higher charge mobility. For instance, a solvent additive of 1% 1,8-diiodoctane (DIO) was employed as the solvent additive to treat the J71:N2200 blends, resulting in more favorable phase separation and domain size. As a result, the corresponding OSCs device achieved an outstanding PCE of 9.34% with an ultrahigh fill factor (FF) of 77.86% [26]. Furthermore, by replacing DIO with diiodomethane in PM6:L8-BO-based OSCs, the energetic difference between the single excited state of L8-BO and the charge transfer state in blend film was effectively reduced, while the microstructure morphology and charge transport of the optimized blend film were not deteriorated, and thereby the open-circuit voltage (*V*_OC_) increased [27].

In this study, the polymers D18 and PYIT-OD were employed as donor and non-fullerene acceptor materials, respectively, for the fabrication of all-polymer solar cells (all-PSCs) using the LBL method. By fine-tuning the mixture of solvents, we systematically study how solvent additives influenced the photovoltaic performance of all-PSCs. The addition of a 2% chloronaphthalene (CN) additive led to a PCE of 15.07%. This study also investigates the impact of different solvent additives on the exciton dynamics, molecular crystallinity, and microstructure morphology within the D18/PYIT-OD-based LBL all-PSCs devices. Our study demonstrated the great potential of additive engineering via LBL fabrication methods in regulating microstructure of active layers, suppressing carrier recombination and enhancing the photovoltaic performance of devices.

## 2. Result and Discussion

**Figure 1:**
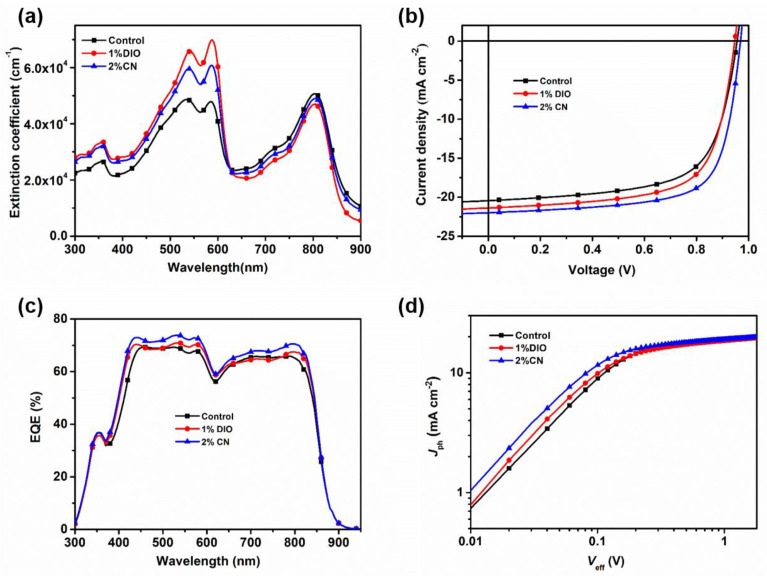
(**a**) The extinction coefficient of the D18/PYIT-OD bilayer film fabricated from different processing conditions; (**b**) the representative *J*-*V* curves of devices at different solvent additives; (**c**) the representative EQE spectra of devices at different solvent additives; (**d**) the *J*_ph_*–V*_eff_ curves of devices at different solvent additives.

The chemical structures of D18 and PYIT-OD are illustrated in Figure S1. Figure 1a shows the extinction coefficient of a D18/PYIT-OD bilayer film fabricated from different processing conditions. The introduction of additives noticeably enhanced the extinction coefficient of the film, with values reaching 4.8 × 10^4^ cm^−1^, 6.9 × 10^4^ cm^−1^, and 6.1 × 10^4^ cm^−1^ under the three different processing conditions. This indicates an improvement in the molecular packing of the film due to the presence of additives. Since 585 nm is the main absorption peak of D18, our main focus is on the changes at this position under different additive conditions. The absorption peak ratio Ι_0–0_/Ι_0–1_ of the D18 film under three different processing conditions rose from 0.99 in the controlled device to 1.06 and 1.02 for bilayer films with DIO and CN additives, suggesting that the D18 film exhibited more uniform π-π stacking with the presence of additives. The increase in crystallinity and the optimization of molecular packing are likely to enhance charge transport and photovoltaic performance. To verify whether the DIO and CN additives could enhance the photovoltaic performance of OSCs, we fabricated devices using the LBL method with a structure of ITO/PEDOT:PSS/D18/PYIT-OD/PDIN/Ag. DIO and CN additives were added to the PYIT-OD solution and measured by volume percentage. Figure 1b and Figure S2 show the representative current density–voltage (*J*-*V*) characteristics of the devices at different concentrations of solvent additives, with the specific photovoltaic parameters presented in Table 1 and Table S1. The PCE for the control device (without any additive) was 12.91% with a short-circuit current density (*J*_SC_) of 20.44 mA cm^−2^, an open-circuit voltage (*V*_OC_) of 0.956 V, and a fill factor (FF) of 66.06%. When a DIO or CN additive was added, the PCE of the devices first increased and then decreased, reaching maximum PCE of 15.07% and 13.72% at 2% CN and 1% DIO concentrations, respectively. The corresponding *J*_SC_ was 22.00 and 21.38 mA cm^−2^, *V*_OC_ was 0.969 and 0.947 V, and FF was 70.71% and 67.73%, all of which were superior to the control devices. In order to validate the accuracy of the *J*-*V* measurements, the external quantum efficiency (EQE) spectra for all devices were measured and illustrated in Figure 1c and Figure S3. The *J*_SC_ values derived from the EQE spectra aligned with those obtained from *J*-*V* measurements. All devices exhibited a wide photo-response range spanning from 400 to 850 nm. In particular, when 2% CN was added into the PYIT-OD solution, the EQE values between 450 and 575 nm and 725 and 825 nm were significantly higher than those of other devices.

To investigate the excitons dissociation and charge collection within devices, we conducted experiments to assess the relationship between photo-generated current (*J*_ph_) and the effective voltage (*V*_eff_), as shown in Figure 1d. Here, the photo-generated current (*J*_ph_) and the effective voltage (*V*_eff_) are calculated by *J*_ph_ = J_L_ − J_D_ and *V*_eff_ = V_0_ − V, respectively, where *J*_L_ and *J*_D_ refer to the current density under illumination and dark conditions, V_0_ is the voltage when *J*_ph_ is zero, and V is the applied voltage [28,29]. Under a large *V*_eff_, the *J*_ph_ tends to be saturated (*J*_sat_), at which point nearly all excitons are dissociated into free carriers and collected by the electrodes. We also analyzed the probability of the charge dissociation (*P*(E,T)) of devices under different processing conditions (Table S2), which was calculated by the ratio of *J*_ph_ to *J*_sat_ under short-circuit conditions. For the devices without additives, with the addition of a 1% DIO additive and a 2% CN additive, the *P*(E,T) values were 89.3%, 91.4%, and 94.9%, respectively. Based on the equation *J*_sat_ = *qG*_max_*L*, we calculated the corresponding maximum exciton generation rates (*G*_max_) was to be 1.31 × 10^28^, 1.32 × 10^28^, and 1.38 × 10^28^ m^−3^ s^−1^, respectively. Devices with a 2% CN additive showed increased *G*_max_, which correlated well with their higher *J*_SC_, potentially contributing to the improved light absorption and more orderly molecular packing. These results demonstrate that devices with 2% CN additive exhibit superior performance in exciton and charge dynamics.

**Figure 2:**
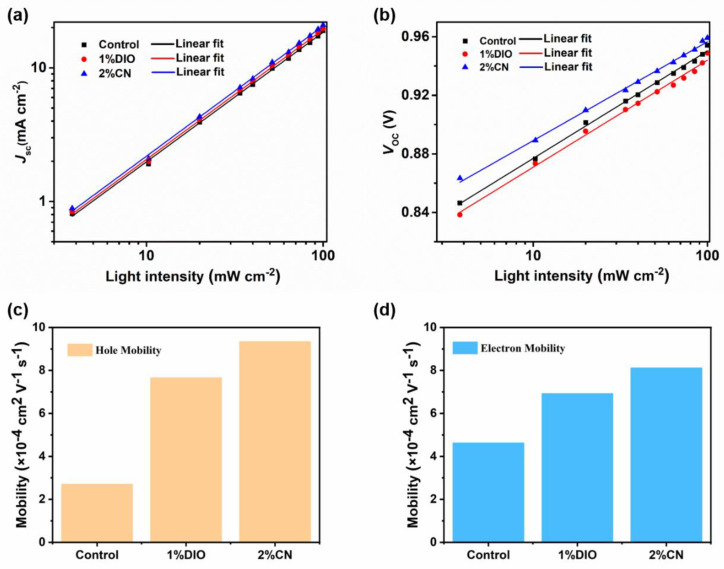
(**a**) The relationship between *J*_SC_ and *P*_light_; (**b**) the relationship between *V*_OC_ and *P*_light_; (**c**) the hole mobility; and (**d**) electron mobility of the devices.

To delve into the charge recombination of different devices, we performed *J*-*V* under various light intensities (*P*_light_) and plotted the correlation curves of *J*_SC_ and *V*_OC_ with *P*_light_. The relationship between *J*_SC_ and *P*_light_ follows the formula *J*_SC_ ∝ (*P*_light_)^S^, where S reflects the intensity of bimolecular recombination in the device. Generally, the S value being very close to the unit suggests that OSC devices experience minimal bimolecular recombination [30]. As shown in Figure 2a, for devices without an additive, with a 1% DIO additive, and with a 2% CN additive, the corresponding S values were 0.96, 0.97, and 0.98, respectively. The results indicate that devices based on D18/PYIT-OD with 2% CN are most effective in suppressing bimolecular recombination. Meanwhile, in the *V*_OC_ versus *P*_light_ curves shown in Figure 2b, the controlled device exhibited the highest fitting slope, suggesting severe trap-assisted Shockley–Read–Hall (SRH) or bimolecular recombination. For the device with 1% DIO additives and 2% CN additives, the slopes were decreased to 1.60 *k*_B_*T*/*q* and 1.52 *k*_B_*T*/*q*, respectively. The smallest fitting slope for the device with the CN additive indicated that SRH or bimolecular recombination is effectively suppressed, promoting effective charge transport and collection.

Single-carrier devices based D18/PYIT-OD bilayer films were fabricated to investigate the charge transport behavior through the space charge-limited current (SCLC) method [31], and the summarized data are listed in Table S3. The specific device structure for the hole-only device is ITO/PEDOT:PSS/D18/PYIT-OD/Ag, while the electron-only device is ITO/ZnO/D18/PYIT-OD/PFN-Br/Ag. As shown in Figure 2c,d, the hole mobility (μ_h_) and electron mobility (μ_e_) of the device without additive are 3.71 × 10^−4^ cm^2^ V^−1^ s^−1^ and 4.63 × 10^−4^ cm^2^ V^−1^ s^−1^, respectively. After adding additives to PYIT-OD, the measured μ_h_ increased to 7.66 × 10^−4^ cm^2^ V^−1^ s^−1^ (1% DIO) and 9.34 × 10^−4^ cm^2^ V^−1^ s^−1^ (2% CN), while the μ_e_ increased to 6.93 × 10^−4^ cm^2^ V^−1^ s^−1^ (1% DIO) and 8.18 × 10^−4^ cm^2^ V^−1^ s^−1^ (2% CN). It is evident that the device treated with the 2% CN additive not only has a more efficient charge transport performance but also a more balanced charge transport, indicating an effective reduction in charge accumulation and recombination, thereby improving the *J*_SC_ of devices. In addition, exciton separation and charge transfer at the electron donor and acceptor interface are crucial for a device’s photovoltaic performance. To investigate the exciton separation and charge transfer of bilayer films, we created steady-state photoluminescence (PL) spectra of the pristine D18 film and the D18/PYIT-OD bilayer films, as shown in Figure S4. The films were excited at 467 nm, and D18 exhibited typical emission peaks at 620 and 680 nm. The PL intensity at these emission peaks was significantly quenched in the D18/PYIT-OD bilayer films, indicating efficient charge transfer at the electron donor and acceptor interface. For the bilayer film with added 2% CN, this PL quenching is most pronounced, indicating the most efficient charge transfer.

**Figure 3:**
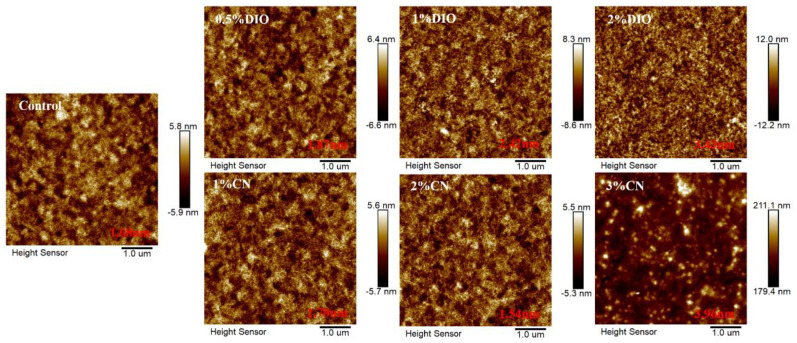
AFM images of bilayer films based on D18/PYIT-OD under different processing conditions.

**Figure 4:**
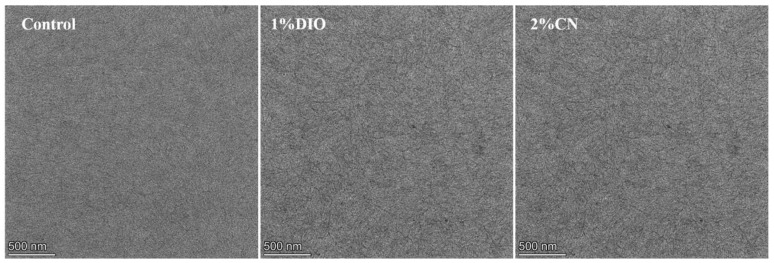
TEM images of bilayer films based on D18/PYIT-OD under different processing conditions.

We used atomic force microscopy (AFM) to observe the influence of the type of additives and their concentrations on the surface morphology of bilayer films. As shown in Figure 3, the root-mean-square (RMS) roughness value is 1.68 nm for the bilayer film without any additives. With an increasing DIO concentration from 0.5% to 2%, the RMS roughness values rose from 1.87 nm to 3.43 nm. When the CN concentration increased from 1% to 2%, the RMS roughness values slightly decreased from 1.70 nm to 1.54 nm, exhibiting a more refined structure. However, an excessive amount of additive (3% CN concentration) led to large-scale aggregation, resulting in a sharp increase in film RMS roughness, which severely damaged device efficiency. Additionally, by examination of the transmission electron microscopy (TEM) images for both the controlled film and those added with 1% DIO and 2% CN (Figure 4), it becomes apparent that these additives precipitate morphological inhomogeneity within the film, likely attributable to the induced crystallization. Comparative analysis indicates that the addition of 2% CN results in a fiber-like film structure, leading to higher FF in the corresponding devices. This also aids in the effective separation of excitons and charge transport.

**Figure 5:**
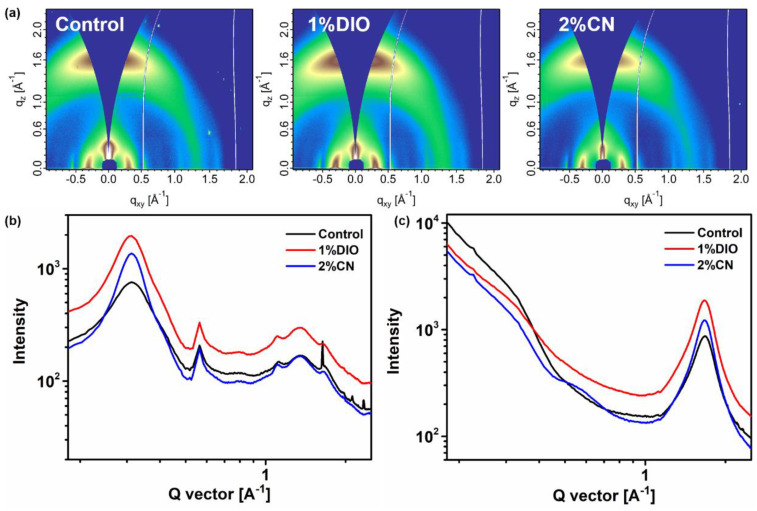
(**a**) Two-dimensional GIWAXS patterns of bilayer films based on D18/PYIT-OD under different processing conditions; GIWAXS line-cuts of bilayer films based on D18/PYIT-OD under different processing conditions (**b**) in the in-plane direction and (**c**) in the out-of-plane direction.

To gain a deeper understanding of the molecular packing and crystalline properties within the active layer, we utilized grazing-incidence wide-angle X-ray scattering (GIWAXS) measurements to examine the D18/PYIT-OD bilayer films under various processing conditions. Figure 5a shows the 2D GIWAXS patterns of D18/PYIT-OD bilayer films without any additive (control), with 1% DIO, and with 2% CN. The corresponding line-cut plots in the in-plane (IP) and out-of-plane (OOP) directions are shown in Figure 5b,c, respectively. From the 2D GIWAXS patterns under three different processing conditions, we observed a strong (010) π-π stacking peak in the OOP direction and a distinct (100) lamellar stacking peak in the IP direction. This indicates that the D18/PYIT-OD bilayer films predominantly exhibited a face-on orientation. This result also implied that the fabrication of the upper layer of PYIT-OD does not significantly alter the molecular orientation of the D18 film [32]. For the bilayer film with 1% DIO, the diffraction peak intensities are more pronounced for both the IP lamellar stacking and the OOP π-π stacking. In addition, it is evident that the bilayer film containing 2% CN exhibits a minimum full width at half maximum (FWHM) value of 0.292 Å at 1.69 Å^−1^ in the OOP direction, indicating strong π-π stacking. In comparison, the FWHM values for the controlled bilayer film and the film with 1% DIO are 0.352 Å and 0.317 Å, respectively. The reduced FWHM value in the film with 2% CN suggests a higher crystal coherence length (CCL). A larger CCL suggested an increase in the number of crystal repeating units within the active layer, which is more conducive to the charge transport. This is consistent with the results above where the OSC device containing 2% CN exhibited the highest hole and electron mobility. The bilayer film that incorporated the 1% DIO additive suffered from excessive aggregation, leading to a compromised photovoltaic performance of the OSC device.

## 3. Experimental Details

### 3.1. Materials

The D18 polymer and the non-fullerene polymer acceptor PYIT-OD were purchased from Derthon Optoelectronics Materials Science Technology Co., Ltd. (Shenzhen, China). Other reagents and solvents were purchased from commercial sources and used without any further treatment.

### 3.2. Device Fabrication

The solution-processed layer-by-layer solar cells devices were fabricated with a conventional structure of Indium tin oxide (ITO)/PEDOT:PSS/D18/L PYIT-OD/PDIN/Ag and the fabrication details are as follows: ITO coated glass substrates were cleaned prior to device fabrication by sonication in acetone, detergent, distilled water, and isopropyl alcohol. After being treated with an oxygen plasma for 20 min, 40 nm thick poly(styrene sulfonate)-doped poly(ethylene-dioxythiophene) (PEDOT:PSS) (Bayer Baytron 4083) layer was spin-casted on the ITO-coated glass substrates at 3000 rpm for 30 s, the substrates were subsequently dried at 150 °C for 10 min in air and then transferred to a N_2_ glovebox. The donor and acceptor were dissolved in chloroform (CF) with the concentration of 5 mg/mL and 7 mg/mL, respectively. The D18 solution was stirred at 100 °C for 30 min, followed by stirring at 40 °C for 2 h. After the D18 had completely dissolved in CF, spin-coating with the speed of 3000 rpm at a fixed temperature of 40 °C was performed on the prepared substrate with PEDOT:PSS. The PYIT-OD solution was stirred at 60 °C for 1 h, and then spin-coated at room temperature. The spin-coating conditions were 4000 rpm for 40 s. The thickness of the active layer was 110 nm. PDIN layer was deposited by spin casting from 2 mg/mL solution in methanol. Finally, Ag (~90 nm) was evaporated with a shadow mask as the top electrode. The effective area was measured to be 0.0516 cm^2^.

### 3.3. Measurements and Instruments

UV-vis-NIR spectra of pure and blend films on a quartz substrate were recorded at room temperature (ca. 25 °C) using a UV-3600 Plus UV-Vis-NIR spectrometer. Photoluminescence spectra were recorded on a Horiba Nanolog fluorescence spectrophotometer. PCEs were determined from *J*-*V* characteristics measured by a Keithley 2400 source-measurement unit under AM 1.5G spectrum from a solar simulator (Oriel model 91192). Solar simulator illumination intensity was determined using a monocrystal silicon reference cell (Hamamatsu S1133, with KG-5 visible color filter) calibrated by the National Renewable Energy Laboratory (NREL). External quantum efficiency (EQE) values of the encapsulated devices were measured by using an integrated system (Enlitech, Kaohsiung City, Taiwan) and a lock-in amplifier with a current preamplifier under short-circuit conditions. The devices were illuminated by monochromatic light from a 75 W xenon lamp. The light intensity was determined by using a calibrated silicon photodiode. Steady-state photoluminescence tests were performed using a Fluorolog 3 spectrofluorometer (HORIBA Instruments Incorporated, Kyoto, Japan). The Transient absorption (TA) spectra were collected by a home-built TA system described briefly as below. The fs laser from an amplifier (800 nm, 1 KHz, Legend Elite F 1K HE+II, Coherent, Saxonburg, PA, USA) was used as the light source. The output from the amplifier (800 nm) or a doubled frequency (400 nm) were employed as the pump light. For the pump light, “on” and “off” were regulated by a mechanical chopper (500 Hz, MC2000B-EC, Thorlabs, Newton, NJ, USA) in the pump beam. The supercontinuum white light generated by a 3 mm thick sapphire plate was used as the probe light, which was then collected by a spectrometer (300 nm–1100 nm, Omni-λ200i, Zolix, Beijing, China). The delay between the probe light and the pump light is controlled by a mechanical delay stage. The atomic force microscopy (AFM) measurements of the surface morphology of D18 films were conducted on a Dimension Icon Scanning Probe Microscope system.

### 3.4. Charge Carrier Mobility Measurement

Hole and electron mobilities were measured by the space-charge limited current (SCLC) method with the hole-only device structure of ITO/PEDOT:PSS/D18/PYIT-OD/Ag and ITO/ZnO/D18/PYIT-OD/PFN-Br/Ag electron-only devices, respectively. The active layers for these devices were spin-coated under the same condition as that of solar cells. *J–V* curves in the range of −4 to 4 V were gained by a Keithley 2400 source-measure unit in dark conditions. The mobilities were obtained by fitting *J–V* curves with the formula of:

*J* = 9*ε_o_ε_r_µV*^2^/(8*L*^3^)

where *J* is the current density, *L* is the thickness of the active layer, *μ* is the mobility, *ε_o_* is the vacuum dielectric constant, *ε_r_* is the relative dielectric constant of the transport medium, and *V* (*V*_app_ − *V*_bi_) is the internal voltage, where *V*_app_ is the applied voltage and *V*_bi_ is the built-in voltage.

## 4. Conclusions

In conclusion, LBL all-PSCs based on the D18/PYIT-OD were fabricated by incorporating solvent additives into the PYIT-OD acceptors to regulate the appropriate microstructure. CN and DIO additives have a significant impact on the molecular packing and morphology of the active layer. With the optimized amount of additives, both CN and DIO can significantly enhance the photovoltaic performance of the devices, particularly CN, which is very effective in improving the *J*_SC_ and FF. The presence of additives also affects the crystallization and packing of D18, thereby affecting the charge mobility of the devices. In addition, devices containing a 2% CN additive experienced a significant increase in exciton separation, and a marked reduction in bimolecular recombination and trap-assisted recombination. Finally, a morphological study confirmed that the presence of additives significantly improves the molecular crystallization and packing of the active layer, resulting in enhanced crystallinity and CCL. As a result, the optimized LBL all-PSCs based on the D18/PYIT-OD delivered a high PCE of 15.07%. The strategy of separately regulating the morphology of the electron donor and acceptor layers via the LBL fabrication method makes it a very promising approach to promote the development of organic photovoltaic technology.

## Figures and Tables

**Table 1:** Summary data of photovoltaic performance of devices with different solvent additives.

Condition	*V_OC_* (V)	*J_SC_*/*J_Cal_* (mA cm^−2^)	FF (%)	PCE (%)
Control	0.956	20.44/19.88	66.06	12.91 (12.62 ± 0.22) ^a^
1% DIO	0.947	21.38/21.00	67.73	13.72 (13.43 ± 0.20) ^a^
2% CN	0.969	22.00/21.50	70.71	15.07 (14.88 ± 0.26) ^a^

^a^ Average PCE values were obtained from 10 devices.

## Data Availability

Data are contained within the article and Supplementary Materials.

## Supplementary Materials

The following supporting information can be downloaded at: https://www.mdpi.com/article/10.3390/molecules29122879/s1, Figure S1 Chemical structures of D18 and PYIT-OD. Figure S2 J-V curves of devices at different solvent additives concentration. Figure S3 EQE curves of devices at different solvent additives concentration. Figure S4 PL spectra of the pristine D18 film and the D18/PYIT-OD bilayer films. Table S1 Photovoltaic parameters for D18/PYIT-OD-based all-PSCs. Table S2 The parameters of exciton dissociation efficiency and *G*max of devices obtained from different processing conditions. Table S3 Summary of the fitting data for hole-only and electron-only devices.
