# Supplementary material for: Layer-by-Layer-Processed All-Polymer Solar Cells with Enhanced Performance Enabled by Regulating the Microstructure of Upper Layer
Source: Molecules. 2024 Jun 17;29(12):2879. doi: 10.3390/molecules29122879 (PMC11206570; doi:10.3390/molecules29122879)
Supplement: Supplementary file 1 [file molecules-29-02879-s001.zip › molecules-3035308-supplementary.pdf]

Supporting information

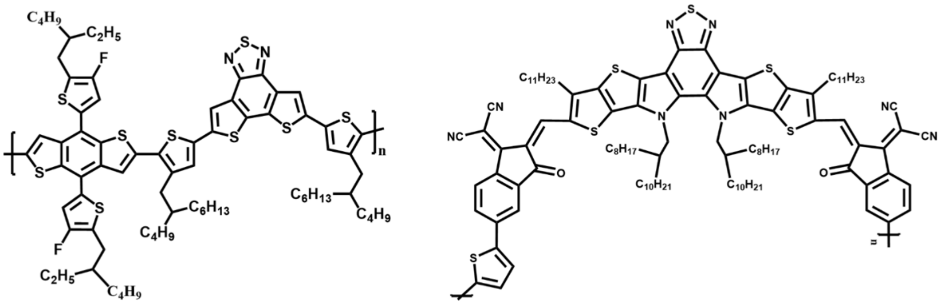

D18 **PYIT-OD**

Figure S1 Chemical structures of D18 and PYIT-OD.

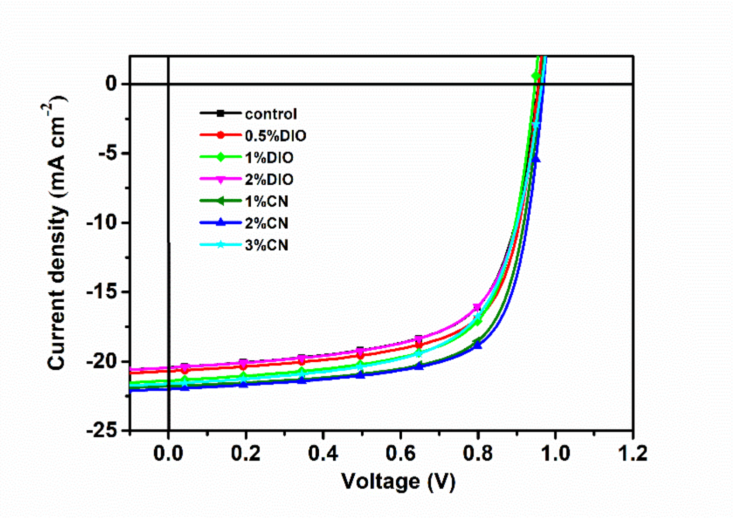

Figure S2 *J-V* curves of devices at different solvent additives concentration.

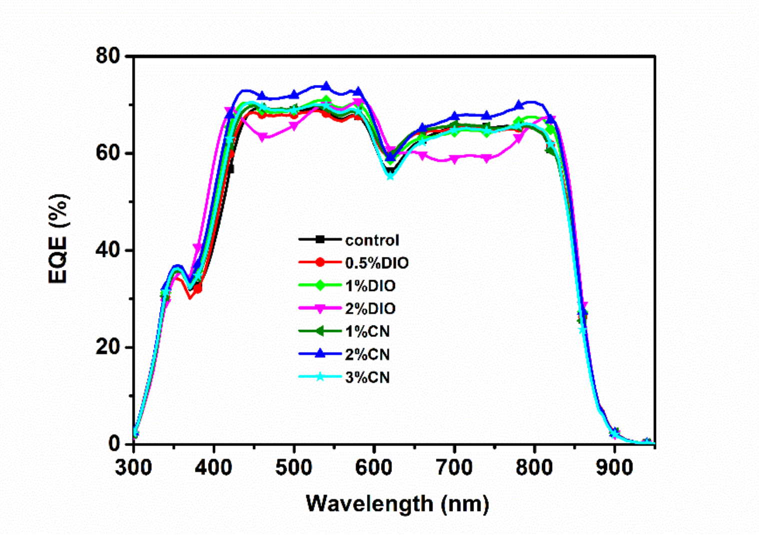

Figure S3 EQE curves of devices at different solvent additives concentration.

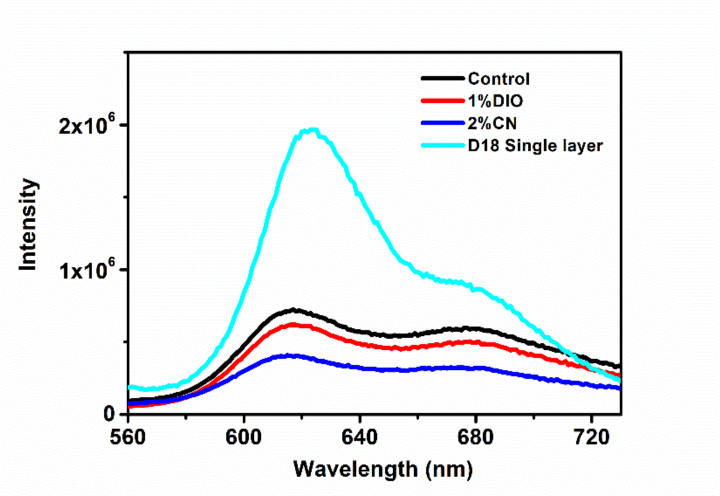

Figure S4 PL spectra of the pristine D18 film and the D18/PYIT-OD bilayer films.

Table S1 Photovoltaic parameters for D18/PYIT-OD-based all-PSCs

Condition	J_{SC} (mA cm^{-2})	V_{OC} (V)	FF(%)	PCE(%)
Control	20.44	0.956	66.06	12.91
0.5%DIO	20.69	0.957	68.28	13.51
1%DIO	21.38	0.947	67.73	13.72
2%DIO	20.44	0.964	65.36	12.89
1%CN	21.78	0.963	70.55	14.80
2%CN	22.00	0.969	70.71	15.07
3%CN	21.59	0.965	65.01	13.54

Table S2 The parameters of exciton dissociation efficiency and G_{max} of devices obtained from different processing conditions

Devices	J_{ph}(mA cm^{-2})	J_{sat} (mA cm^{-2})	η_{diss}(%)	G_{max}(10^{28} m^{-3} s^{-1})
Control	20.65	23.12	89.3	1.31
1% DIO	21.38	23.40	91.4	1.32
2% CN	22.97	24.20	94.9	1.38

Table S3 Summary of the fitting data for hole-only and electron-only devices.

Devices	μ_h (10^{-4} cm^2 V^{-1} s^{-1})	μ_e (10^{-4} cm^2 V^{-1} s^{-1})	μ_h/μ_e
Control	3.71	4.63	0.80
1% DIO	7.66	6.93	1.11
2% CN	9.34	8.18	1.14
